# Brain Connectivity Reflected in Electroencephalogram Coherence in Individuals With Autism: A Meta-Analysis

**DOI:** 10.32598/bcn.9.10.375

**Published:** 2019-09-01

**Authors:** Vida Mehdizadefar, Fanaz Ghassemi, Ali Fallah

**Affiliations:** 1. Department of Biomedical Engineering, School of Electrical, Computer & Biomedical Engineering, Amirkabir University of Technology, Tehran, Iran.

**Keywords:** Autism spectrum disorder, Electroencephalography, Coherence, Meta-analysis

## Abstract

**Introduction:**

Many theories have been proposed about the etiology of autism. One is related to brain connectivity in patients with autism. Several studies have reported brain connectivity changes in autism disease. This study was performed on Electroencephalogram (EEG) studies that evaluated patients with autism, using functional brain connectivity, and compared them with typically-developing individuals.

**Methods:**

Three scientific databases of ScienceDirect, Medline (PubMed), and BioMed Central were systematically searched through their online search engines. Comprehensive Meta-analysis software analyzed the obtained data.

**Results:**

The systematic search led to 10 papers, in which EEG coherence was used to obtain the brain connectivity of people with autism. To determine the effect size, Cohen’s d parameter was used. In the first meta-analysis, the study of the maximum effect size was considered, and all significant effect sizes were evaluated in the second meta-analysis. The effect size was assessed using a random-effects model in both meta-analyses. The results of the first meta-analysis indicated that heterogeneity was not present among the studies (Q=13.345, P>0.1). The evaluation of all effect sizes in the second meta-analysis showed a significant lack of homogeneity among the studies (Q=56.984, P=0.0001).

**Conclusion:**

On the whole, autism was found to be related to neural connectivity, and the present research showed the difference in the EEG coherence of people with autism and healthy people. These conclusions require further studies with more extensive data, considering different brain regions, and novel analysis techniques for assessing brain connectivity.

## Highlights

- Autism spectrum disorders change functional brain connectivity.
- EEG coherence in people with autism differs from healthy people.
- Connectivity can be an appropriate biological marker for the early diagnosis of autism.

## Plain Language Summary

Autism spectrum disorders are neurodevelopmental disorders with unknown cause, affecting the normal functioning of the brain. According to some studies, it may be related to aberrant brain connectivity. This review study compares the results of all these papers to determine whether brain connections are different in autistic and healthy individuals. Eventually, autism was found to be related to neural connectivity, and the present research showed the difference in the coherence (a simple connectivity measure) of people with autism and healthy ones. According to this result, connectivity can be an appropriate biological marker for the early diagnosis of autism. The golden age for identifying and treating autism is a short period between 2 and 5 years old. If the training and treatment interventions are not carried out within this short period, the next steps will not yield much; therefore, the prevention and early detection of this disorder will be of great importance.

## 1. Introduction

Autism Spectrum Disorder (ASD) is a neurobehavioral condition that changes the normal brain function. It is characterized by impairments in social interaction, speech, and non-verbal communication, eye contact, repetitive behaviors, group activities, and imagination (Kanner, 1943; Landa, 2008). The prevalence rate of autism in the United States has risen from 1 in 150 children in 2006 to 1 in 68 in 2014. According to the newest report of the Center for Disease Control and Prevention, it remained unchanged until 2016 (Port et al., 2016). The prevalence of autism was less than three per 10000 individuals in the 1970s and rose to more than 30 per 10000 in the 1990s. This rate is a 10-time increase for 20 years that imposes a high cost on society. So, the assessment of autistic individuals is an important issue (Blaxill, 2004).

Since the introduction of autism, many studies have been conducted to assess brain functions in individuals with autism. The main research areas are the genetics of autism, brain networks involved in the incidence of this disorder, looking for the appropriate biological markers for early diagnosis, as well as measuring brain connectivity. Studies showed that individuals with autism have different brain connectivity patterns compared with typically-developing groups. A leading theory of ASD suggests that autism may occur because of the aberrant neural connectivity patterns (Cantor, Thatcher, Hrybyk, & Kaye, 1986; Mak-Fan et al., 2013; Minshew & Williams, 2007). Evidence in support of this theory was based on the investigations of Positron Emission Tomography (PET), Magnetic Resonance Imaging (MRI), and EEG, as well as microscopic research after death (Coben & Myers, 2008). Abnormal neural connectivity results in different levels of processing in brain networks and, therefore, deficits in the neural and cognitive integration of information (Hernandez, Rudie, Green, Bookheimer, & Dapretto, 2015). Autism has a strong genetic basis with a highly heritable nature; so, changes in the functional and structural connectivity are possible phenotypes for this disease and maybe an essential aspect of the ASD profile (Moseley et al., 2015).

Connectivity groups into 2 major structural and functional types. Structural connectivity denotes physical connections, usually assessing by fiber tractography. Functional connectivity refers to the statistical dependencies between neurophysiological events, which are spatially independent. Different tools can be utilized to measure brain connectivity. Diffusion tensor imaging and MRI are conventional methods for measuring structural connectivity that represent fibers within brain networks. Functional connectivity can be achieved by utilizing imaging techniques such as functional MRI (fMRI) or other measures of brain activity such as EEG and Magnetoencephalography (MEG). The investigation of brain connectivity using EEG has advantages over other devices because of its significant lower cost, availability, and high precision time measurements. Thus, EEG is a suitable tool for describing the dynamic activation and deactivation of functional networks and their connectivity. This review will focus on studies that measure the connectivity, using EEG signals in patients with ASD and typically-developing individuals.

A simple measure of connectivity, linear coherence, has been evaluated in most EEG studies. It was first used for representing the connectivity impairments of autism in the 1980s (Cantor et al., 1986). The coherence measure is a function of frequency and explains synchronization between 2 EEG signals of the same frequency. Many papers in the field of autism focus on connectivity issues and report different results. Many reasons justify these differences, such as theoretical models, the measurement procedure, and participants’ characteristics. In the following, we refer to the studies used coherence and obtained heterogeneous results. In autistic participants, the connectivity between 2 hemispheres during visual tasks is assessed (Isler, Martien, Grieve, Stark, & Herbert, 2010). In this study, coherence measures within the occipital region and between hemispheres were examined; the results indicated that the EEG coherence between 2 hemispheres in individuals with autism was low. In another research (Catarino et al., 2013), inter-hemispheric coherence was evaluated, using wavelet coherence, in which children with ASD represented reduced inter-hemispheric coherence.

Reduced connectivity between long-range distances have appeared also in task-free studies (Barttfeld, Wicker, Cukier, Navarta, & Lew, 2011; Cantor et al., 1986; Coben & Myers, 2008; Duffy & Als, 2012; Lazarev, Pontes, & Mitrofanov, 2010; Murias, Webb, Greenson, & Dawson, 2007). In the low-frequency bands (delta and theta), weaker coherence between frontal and occipital brain regions has been reported (Barttfeld et al., 2011; Lazarev et al., 2010). In contrast, increased coherence in the theta band and reduced coherence in the alpha band have been found among the frontal and the temporal, parietal, and occipital regions (Murias et al., 2007). In another study, decreased connectivity in the beta band has been reported between frontal and temporal regions (Duffy & Als, 2012). Investigations on short-range connections in resting-state EEG studies are less consistent. Intra-hemispheric and inter-hemispheric connections in all brain regions have claimed to decrease in the delta and theta bands (Lazarev et al., 2010). Coherence over the frontal area has been reduced in the delta and alpha bands (Barttfeld et al., 2011; Murias et al., 2007). However, increased coherence has been shown within the frontal region in the delta band (Barttfeld et al., 2011), and within frontal and temporal regions in the theta band (Murias et al., 2007).

A systematic review of EEG and MEG studies has demonstrated reduced long-range connectivity in individuals with ASD compared to the controls (O’Reilly, Lewis, & Elsabbagh, 2017). In this paper, because of the different modalities and connectivity metrics, quantitative analysis was not done.

There are differences in the results of brain connectivity evaluation across studies. Different outcomes result from the age of patients participating in the study, the brain regions considered, and the frequency bands in which connectivity was analyzed. So, studies show different brain connectivity patterns in autistic and typically-developing individuals. Many essential factors can influence the results of coherence analyses such as sample characteristics, EEG reference, frequency band, task/resting state, and brain regions. Also, it has some pitfalls (e.g. particular susceptibility to volume conduction, the choice of reference of electrode montage, and coherence estimator) that are beyond the scope of this paper. Nevertheless, the papers that used EEG to study the effects of autism on functional brain connectivity reflected in coherence are outlined in this meta-analysis. A meta-analysis is a technique in the statistical field that considers the results of multiple scientific studies. Thus, the accumulation of data results in better statistical power and a more robust point estimate compared to using individual papers to extract the outcome measures.

## 2. Methods

### 2.1 Search strategy

Relevant papers through a 5-step procedure of search and inclusion/exclusion criteria were selected in the current meta-analysis. Steps are listed in Table 1. Based on the aim of this paper, a literature search was done on ScienceDirect, PubMed, and BioMed Central for papers evaluating EEG coherence associated with autism. Studies published after April 2016 were not included. The following search terms were used in this search: [Autism Spectrum Disorder OR ASD OR Autism] AND [EEG OR electroencephalogram] AND [Brain connectivity OR Connectivity] AND [Functional or Coherence]. At this step, 140 English papers were found.

**Table 1.** Five-step inclusion criteria for the studies reviewed in the meta-analysis

**Steps**	**Inclusion Criteria**
Literature search	ScienceDirect, PubMed, and BioMed Central (1980–2016) Search terminology: “Autism Spectrum Disorder” OR “ASD” OR “Autism” AND “brain connectivity” OR “functional connectivity” OR “coherence” AND “EEG” and any other derivatives of these words
Language	English
Review of titles and abstracts	EEG, autism (any version) and connectivity, or coherence, no reviews, no studies of medication effects, no neurofeedback, no infants, no single case studies
Review of full papers	Coherence (any version) variables, analysis of data in terms of autism vs. controls (must have controls for relative comparison grouped by autism vs. controls), studies with other disorders, not autism, were excluded
Quality of data	The eligibility of the study describes the source of subjects, Randomized Clinical Trials (RCT) studies, the results of between-group statistical comparisons are reported for at least one outcome

### 2.2. Study selection

Titles and abstracts were reviewed to determine whether the studies included autism, EEG, and connectivity. Studies that did not involve EEG and coherence were excluded. Of 140 papers, 32 remained. Then, the full text of the papers was reviewed for reporting the results of coherence estimation and the analysis of data in terms of autism versus typically-developing groups. Studies without controls or groups compared to subjects with other disorders, not autism, and those without sufficient information about the output were excluded. Review articles were excluded, too. After applying all these criteria, 12 papers remained.

**Figure 1.**
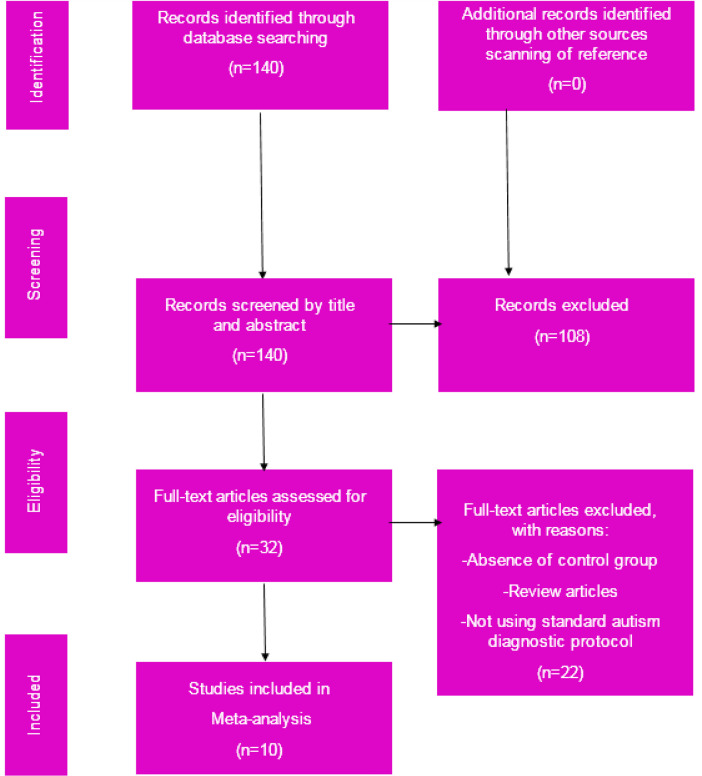
Preferred reporting items for systematic reviews and meta-analysis flowchart for the selection procedure of articles

The last step of the inclusion/exclusion criteria was verifying the quality of data and study eligibility. Only those studies that reported the source of subjects and used standard autism diagnostic protocol were included. Studies that did not involve at least one between-group statistical comparison were excluded. If the data sets repeated in some studies, the research, which contained the pretty most completed set, would be included, and the others would be excluded. After this step, 10 papers remained for the current meta-analysis. Figure 1 shows the selection flowchart.

### 2.3. Statistical analysis

In the meta-analysis of these studies, the standardized difference in mean was computed as the effect sizes for the coherence of autism versus controls. Effect sizes were calculated based on either study sample size (N), Mean, and Standard Deviation (SD) or statistical data such as T-value and F-value. Since the variety of conditions such as different frequency bands, brain regions, and hemispheres have been considered in the studies, the effect sizes for all of them were taken into account in this research. Effect sizes were reported for studies, using weights assigned to them. Confidence Interval (CI) of 95% were calculated, using standard approaches. The analysis of heterogeneity was applied, using the Q-statistic to inspect the differences among the studies.

The comprehensive meta-analysis software was used to perform a random-effect meta-analysis. Cohen’s d and its related variance were computed for the outcome of each study.

## 3. Results

Ten studies met the inclusion criteria and were included in the meta-analysis. Table 2 presents information on these studies. A total of 26 effect sizes were computed for all conditions considered in studies. The effect size results (Cohen’s d) ranged from 0.001 to 2.701, and the average was (Mean±SD=0.722±0.12). The difference of coherence measure in the left hemisphere in the beta frequency band had the most significant effect size (d=2.701) (Lazarev et al., 2010). The smallest effect size was related to the difference of coherence measure in the left prefrontal region and eyes-open condition (Mathewson et al., 2012).

**Table 2.** Details of the studies included in this meta-analysis

**First Author and Date**	**Brain Region (Frequency Band)**	**Coherence**	**Participants ASD/TD**	**Cohen’s d (Max)**
Chan et al. (2011)	Short- and long-range fronto-posterior (Theta)	Enhanced	21/21	1.239
Mathewson et al. (2012)	Short intrahemispheric, posterior (Alpha)	Reduced	15/16	1.412
	long-range intrahemispheric, frontocentral (Alpha)	Reduced		
Lzarev et al. (2010)	Intrahemispheric, frontocentral (entire frequency range)	Reduced	18/14	0754
Lzarev et al. (2010)	Short- and long-range (Delta & Theta)	Reduced	6/8	2.701
	Frontal-temporal (Delta)	Enhanced		
	Frontal-temporal (Theta)	Enhanced		
Léveille et al. (2010)	Short- and long-range intrahemispheric, Occipital (entire frequency range)	Enhanced	9/13	0.923
	Short- and long-range intrahemispheric, Frontal (entire frequency range)	Reduced		
Duffy & Als (2012)	Short- and long-range intrahemispheric (Theta)	Reduced	447/99	0.842
	Short- and long-range intrahemispheric (Alpha)	Reduced		
	Frontal-temporal (Beta)	Reduced		
	Short- and long-range intrahemispheric (Beta)	Reduced		
Coben & Mayers (2008)	Short- and long-range intrahemispheric (Delta & Theta)	Reduced	20/20	0.949
	Interhemispheric Frontal (Delta & Theta) Temporal (Delta & Theta) Central/parietal/occipital (Delta & Theta)	Reduced		
	Interhemispheric Temporal (Alpha)	Reduced		
	Interhemispheric Central/parietal/occipital (Beta)	Reduced		
Yeung et al. (2014)	Short- and long-range, fronto- posterior, and frontal (Theta)	Reduced	18/18	0.727
Sheikhani et al. (2012)	Short- and long-range intrahemispheric, Frontal-temporal, and Frontal-Central	Reduced	17/11	1.793
	Temporal (Gamma)	Enhanced		
Barttfeld et al. (2011)	Lateral-frontal intrahemispheric (Delta)	Enhanced	10/10	1.910
	Middle frontal (Delta)	Reduced		
	Occipital (Delta)	Reduced		

ASD= Autism spectrum disorder; TD= Typically-developing children

**Figure 2.**
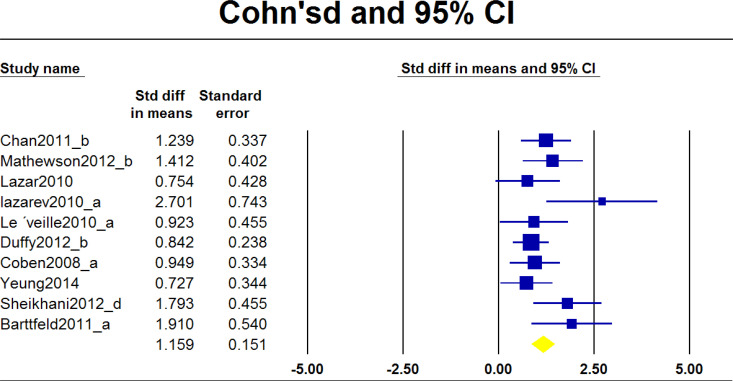
Forest plot considering the most significant effect size for each study

**Figure 3.**
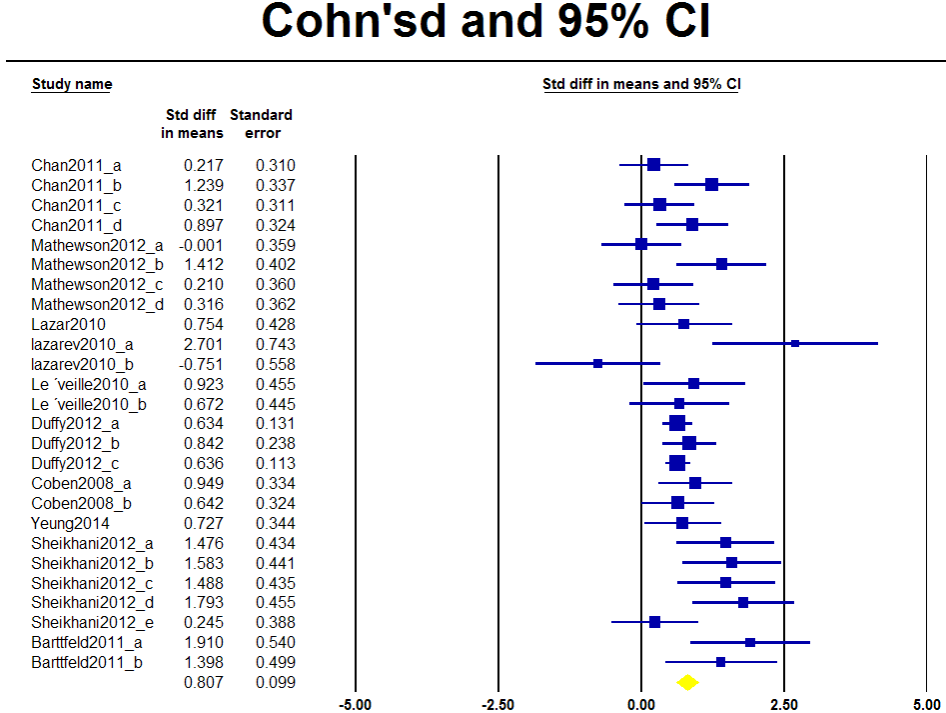
Forest plot considering all effect sizes of studies

The largest effect sizes in each study were considered in the first meta-analysis (Q=13.345, P=0.148) (Figure 2, Table 3). This analysis indicated no significant difference between effect sizes in the studies. The second analysis examined all of the effect sizes reported in each study. A significant difference between effect sizes in the studies was revealed (Q=56.984, P=0.0001) (Figure 3, Table 3).

**Table 3.** Results of random-effects meta-analysis comparing the relative difference in the impact of variants on functional connectivity

**estimates**	**Effect Size and 95% CI**			**Heterogeneity**			**Tau Squared**	

	**Number of Studies**	**Point Estimate**	**Variance**	**Q value**	**P**	**I-Squared**	**Tau Squared**	**Variance**
Maximum estimate	10	1.159	0.023	13.345	0.148	32.56	0.071	0.011
All estimates	26	0.807	0.010	56.984	0.000	56.128	0.119	0.005

## 4. Discussion

The hypothesis of the current meta-analysis is considering studies that investigate ASD-related changes in connectivity. Coherence was accounted as a linear measure of connectivity that is based on the similarity of activations in different regions. The current study aimed to probe the effect of ASD on the functional connectivity reflected in EEG coherence.

Comparing the connectivity in patients with autism and typically-developing controls showed that altered neural connectivity is associated with autism. Two steps of the meta-analysis of Cohen’s d data showed that when the largest effect size of each study was considered, the estimated heterogeneity and I-squared statistics would not reject the null hypothesis in favor of the alternative (i.e. the heterogeneity of studies). Thus, the result (Q=13.345, P=0.148) indicates that heterogeneity is not present among the included studies (Table 3). It would not lead to a conclusive homogeneity because of the small number of studies.

It can be interpreted from the obtained results that the included articles are suitable for the estimation of the single underlying effect size, and within-study variances described the low variance between studies. However, when all effects were taken into account, estimated heterogeneity and I-squared statistics showed that the null hypothesis would be rejected in favor of the alternative (Q=56.984, P=0.0001), demonstrating that heterogeneity was present among the included studies (Table 3). Thus, further exploring the studies for potential sources of heterogeneity such as heterogeneous in the autism spectrum, age patterns, and the brain regions considered may be helpful. Altogether, heterogeneity analysis supports that the studies, despite their prominent differences, are compatible with meta-analysis.

## Ethical Considerations

### Compliance with ethical guidelines

All ethical principles were considered in this article.
